# Phenolic Compounds in Extracts of *Hibiscus acetosella* (Cranberry Hibiscus) and Their Antioxidant and Antibacterial Properties

**DOI:** 10.3390/molecules25184190

**Published:** 2020-09-12

**Authors:** Jae Il Lyu, Jaihyunk Ryu, Chang Hyun Jin, Dong-Gun Kim, Jung Min Kim, Kyoung-Sun Seo, Jin-Baek Kim, Sang Hoon Kim, Joon-Woo Ahn, Si-Yong Kang, Soon-Jae Kwon

**Affiliations:** 1 Advanced Radiation Technology Institute, Korea Atomic Energy Research Institute, Jeongup 56212, Korea; jaeil@kaeri.re.kr (J.I.L.); jhryu@kaeri.re.kr (J.R.); chjin@kaeri.re.kr (C.H.J.); dgkim@kaeri.re.kr (D.-G.K.); jmkim0803@kaeri.re.kr (J.M.K.); jbkim74@kaeri.re.kr (J.-B.K.); shkim80@kaeri.re.kr (S.H.K.); joon@kaeri.re.kr (J.-W.A.); 2 Department of Life-Resources, Graduate School, Sunchon National University, Suncheon 57922, Korea; 3 Division of Plant Biotechnology, College of Agriculture and Life Science, Chonnam National University, Gwangju 61186, Korea; 4 Jangheung Research Institute for Mushroom Industry, Jangheung 59338, Korea; astragali@daum.net; 5 Department of Horticulture, College of Industrial Sciences, Kongju National University, Yesan, Chungnam Province 32439, Korea; sykang@kongju.ac.kr

**Keywords:** *Hibiscus acetosella*, phenolic compound, antioxidant, antibacterial, UPLC

## Abstract

Hibiscus species are rich in phenolic compounds and have been traditionally used for improving human health through their bioactive activities. The present study investigated the phenolic compounds of leaf extracts from 18 different *H. acetosella* accessions and evaluated their biofunctional properties, focusing on antioxidant and antibacterial activity. The most abundant phenolic compound in *H. acetosella* was caffeic acid, with levels ranging from 14.95 to 42.93 mg/100 g. The antioxidant activity measured by the ABTS assay allowed the accessions to be classified into two groups: a high activity group with red leaf varieties (74.71–84.02%) and a relatively low activity group with green leaf varieties (57.47–65.94%). The antioxidant activity was significantly correlated with TAC (0.933), Dp3-Sam (0.932), Dp3-Glu (0.924), and Cy3-Sam (0.913) contents (*p* < 0.001). The *H. acetosella* phenolic extracts exhibited antibacterial activity against two bacteria, with zones of inhibition between 12.00 and 13.67 mm (*Staphylococcus aureus*), and 10.67 and 13.33 mm (*Pseudomonas aeruginosa*). All accessions exhibited a basal antibacterial activity level (12 mm) against the Gram-positive *S. aureus*, with PI500758 and PI500764 exhibiting increased antibacterial activity (13.67 mm), but they exhibited a more dynamic antibacterial activity level against the Gram-negative *P. aeruginosa*.

## 1. Introduction

*Hibiscus acetosella*, a member of the Malvaceae family, is an amphidiploid plant native to Africa and is usually consumed as a green vegetable. In the traditional medicine of western and central Africa, decoction drinks have been prepared from extracts of the leaves and shoots because of their anti-anemic and antipyretic properties [1]. The presence of a wide variety of biochemical compounds, such as polyphenols, flavonoids, and anthocyanins have been reported in Hibiscus species [2,3]. Two examples, *H. cannabinus* and *H. sabdariffa*, have been studied most regarding the relationship between their biochemical compounds in the plant leaves and their biofunctional activity [4,5,6,7], while *H. acetosella* has been much less studied.

Phenolic compounds found in Hibiscus plants consist of phenolic acids such as hibiscus, protocatechuic, gallic, chlorogenic and caffeic acids, and the organic acid, citric acid. Flavonoids such as kaempferitrin, gallocatechin, quercetin, and luteolin are also present in these plants. Anthocyanins, detected mostly in flowers with calyces and some of the red-colored leaves, include cyanidin-3-glucoside, delphinidin-3-galactoside, delphinidin-3-glucoside, cyanidin-3-sambubioside, and delphinidin-3-sambubioside [8,9,10]. These compounds possess antioxidant properties shown by their effective scavenging activity on reactive oxygen species (ROS) and free radicals. Generally, to investigate the antioxidant activity of Hibiscus plants based on phenolic compound levels, the 2,2′-azino-bis (3-ethylbenzthiazoline-6-sulfonic acid) (ABTS) hydroxyl radical scavenging assay and 2,2-diphenyl-1-picryl-hydrazyl-hydrate (DPPH) free radical assay have been widely used [4,7,11]. Kapepula et al. [12] investigated the ability of *H. cannabinus*, *H. sabdariffa*, and *H. acetosella* to scavenge free radicals and reported IC_50_ values ranging from 43 to 186 μg/mL from ABTS and DPPH assays, although the three species differed in the composition of their phenolic compounds.

In terms of pharmacological effects, Hibiscus plants have also attracted interest because of their biological activities, which include antibacterial, anti-inflammatory, antigenotoxic, hepatoprotective, and antimutagenic activities [13,14,15,16]. In particular, the antibacterial activity of *H. sabdariffa* has been well-studied as well as other biological activities of extracts of its leaves and flowers [4,17,18], while *H. acetosella* has been reported as having anti-inflammatory [12] and antimutagenic activity [16], but no antibacterial activity.

The present study therefore aimed to determine the composition of phenolic compounds in the extracts of leaves from 18 *H. acetosella* accessions using UPLC analysis and to assess their bioactive properties, antioxidant and antibacterial activities, using the ABTS assay and agar-well diffusion test, respectively. The relationship between phenolic extracts and their biofunctional properties will also be evaluated using statistical analysis.

## 2. Results and Discussion

### 2.1. Phenolic Composition of H. acetosella Leaf Extracts

**Figure 1:**
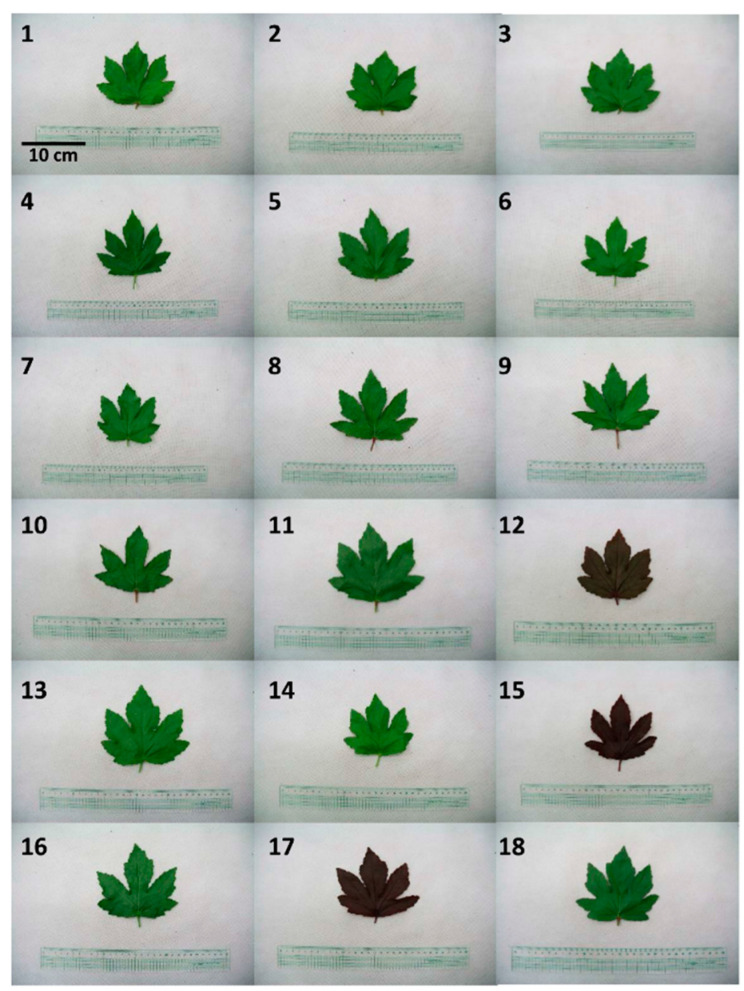
Leaf color of the 18 different *H. acetosella* accessions used in this study.

First, before measuring the content of phenolic compounds, the color characteristics of the *H. acetosella* leaves were investigated (Figure 1). The leaf color is usually green, but three accessions appeared red (PI500777, PI500801, and PI500805). The petiole colors could be divided into four types: 7 accessions were green, 5 accessions green-red, 2 accessions light-red, and 4 accessions red (Table 1). The phenolic compounds of the *H. acetosella* accessions were detected by UV-spectrophotometry and UPLC (Table 2). The total phenolic content (TPC) and total flavonoid content (TFC) levels ranged from 193.14 to 434.67, and 199.10 to 262.19 mg/100 g, respectively, with the highest level in PI500707. A previous study on *H. acetosella*, investigating a different variety using a different extraction method, reported TPC and TFC levels of 1730 and 775 mg/100 g, respectively, values more than three times those found in the present study [19]. Phenolic compounds play an important role in the adaptation of plants to the environment, and their content is determined by the origin, harvesting time, and cultivation conditions [3,20]. Previous data has shown that the TPC and TFC contents can vary by approximately three times according to the region where the plants are collected [21,22]. In the present study, the major polyphenols in *H. acetosella* were identified as caffeic acid (CA) and chlorogenic acid (CGA), while gallocatechin (GC) and gallic acid (GAL) were present in relatively small amounts (Table 2). In particular, CA is present in three Hibiscus plants, *H. cannabinus*, *H. sabdariffa*, and *H. acetosella*, with the highest amounts in *H. acetosella* [12]. CGA is also a major phenolic compound in *H. sabdariffa* aqueous extract [23]. The PI500707 accession also exhibited significantly higher levels of polyphenols such as GC (1.57 mg/100 g), GAL (1.97 mg/100 g), CGA (41.56 mg/100 g), and CA (42.93 mg/100 g) in the collected accessions. As expected, the TAC level was associated with the coloration of the leaf, as well as with the anthocyanins content. In green leaves, the TAC content was less than 1 mg/100 g, but in three accessions with red leaves, the contents ranged from 17.25 to 19.98 mg/100 g (Table 2). Three anthocyanins, delphinidin-3-sambubioside (Dp3-Sam), delphinidin-3-glucoside (Dp3-Glu), and cyanidin-3-sambubioside (Cy3-Sam), were only detected in red leaves but also seen in UPLC 3D profiling (Figure S1). Similar findings have also been reported in a previous study of phenolic compounds in Hibiscus plants [4,6,24].

### 2.2. Effects of Phenolic Extracts on the Radical Cation Scavenging Activity

To compare the antioxidant properties of the 18 *H. acetosella* accessions, the radical cation scavenging activity of their phenolic extracts (10 times diluted) was determined using the ABTS assay (Table 3). The ABTS assay mainly depends on hydrogen peroxide in the presence of ABTS to produce the radical cation and has been previously used for measuring the total antioxidant activity in a wide variety of plants rich in polyphenols [25]. The antioxidant activity varied from the highest value of 84.02% (PI500805) to the lowest value of 57.47% (PI500756) in the *H. acetosella* extracts, with that of the control ascorbic acid (500 µM) at 99.83%. These values of antioxidant activity were higher than those previously reported for *H. acetosella* [26]. The activity of the phenolic compounds from the ABTS assay increased with the contents of the related anthocyanins, TAC, Dp3-Sam, Dp3-Glu, and Cy3-Sam (Table 3). For example, PI500801 (74.71%), PI500777 (82.34%) and PI500805 (84.02%) exhibited the highest antioxidant activity, as well as abundant TAC and anthocyanins contents. In contrast, 15 *H. acetosella* accessions with no detectable anthocyanins contents, showed a slightly decreased antioxidant activity from 57.47% (PI500756) to 65.94% (PI500707). The antioxidant effect of the extracts was probably caused by flavonoids and anthocyanins, especially anthocyanins, which have been reported to exhibit excellent antioxidant activity in Hibiscus plants [27,28]. Interestingly, Maciel et al. [29] have purified anthocyanins such as Dp3-Sam, Dp3-Glu, Cy3-Sam, and cyanidin-3-glucoside (Cy3-Glu) from a crude extract of the *H. sabdariffa* calyx, which exhibited a higher antioxidant activity from the DPPH assay than that of the crude extract. The present study attempted to make use of the DPPH radical activity assay, but an unknown precipitate was produced in the reaction mixture, which affected the measurements in the extracts from some of the accessions. This might have been caused by the reaction of unknown compounds with the organic solvent (Table S1). Nonetheless, all accessions, except for the four aberrant accessions, tended to exhibit a higher antioxidant activity measured by the DPPH assay than that measured by the ABTS activity assay. The antioxidant and bioactive properties of anthocyanins have also been linked to health benefits such as anti-cancer, anti-inflammatory, and anti-diabetic activities [30,31,32,33,34]. Consequently, these results are consistent with previous studies, indicating that anthocyanin compounds were the main contributors to the antioxidant activity of *H. acetosella*.

### 2.3. Inhibitory Effects of Phenolic Extracts against Gram-Positive and Gram-Negative Bacteria

The inhibitory effects of the phenolic extracts of *H. acetosella* leaves on the Gram-positive (*Staphylococcus aureus* ATCC 6538) and Gram-negative (*Pseudomonas aeruginosa* ATCC 9027) bacteria are shown in Table 3. Distilled water, used as the negative control, exhibited no inhibitory effect against the two bacteria (Figure S2), but gentamicin (5 μg), used as the positive control, exhibited inhibition zones against the two bacteria of 12.80 ± 0.34 mm (*S. aureus*) and 13.10 ± 0.42 mm (*P. aeruginosa*). The phenolic extracts of the 18 *H. acetosella* accessions showed antibacterial activity against two bacteria, the zones of inhibition ranging from 12.00 to 13.67 mm (*S. aureus*) and from 10.67 to 13.33 mm (*P. aeruginosa*). For the *S. aureus* bacteria, all accessions exhibited a basal antibacterial activity level (12 mm), with PI500758 and PI500764 exhibiting an increased level of antibacterial activity (13.67 mm), but the Gram-negative (*P. aeruginosa*) bacteria exhibited a wider range of levels of antibacterial activity (Table 3). These antibacterial activities were exhibited similar levels against both Gram-positive and Gram-negative bacteria in *H. sabdariffa* [4,31,35]. The present study first confirmed the presence of antibacterial activity against two bacteria among the phenolic extracts of the 18 different *H. acetosella* accessions as a basis for optimization in future research using a formal antibacterial measuring method.

### 2.4. Relationship between Phenolic Extracts and Biofunctional Properties

**Figure 2:**
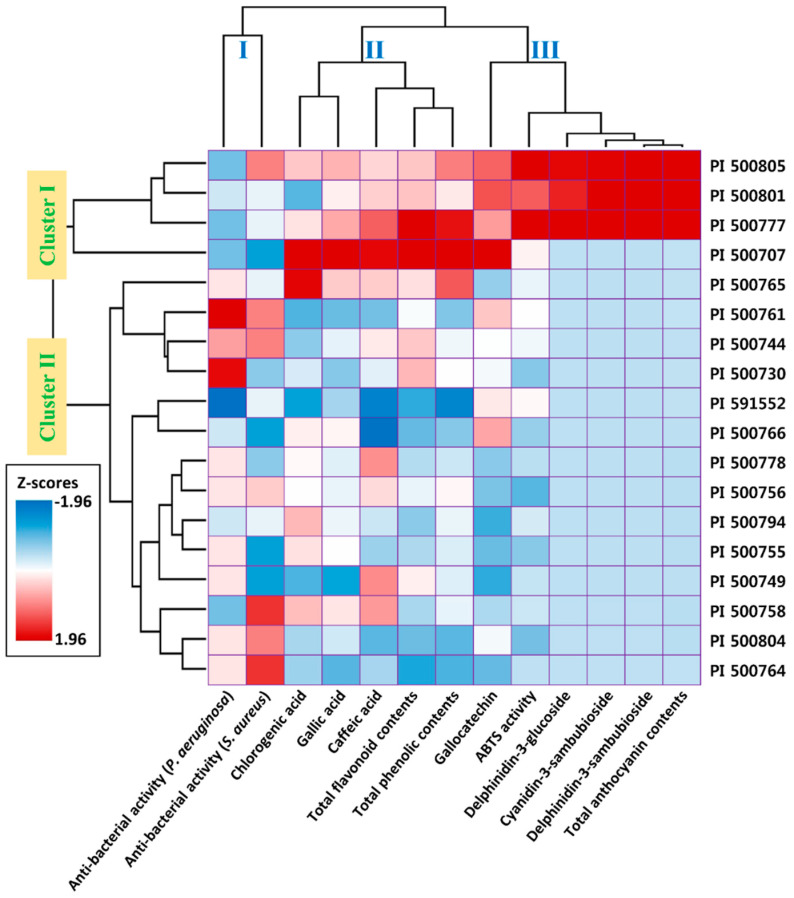
Hierarchical cluster analysis of the 18 different *H. acetosella* accessions according to their phenolic compound contents and biofunctional properties.

The Pearson correlation coefficient and hierarchical clustering were used to assess the relationship between the contents of phenolic compounds in the extracts and the biofunctional properties (antioxidant and antibacterial activities) in the 18 *H. acetosella* accessions (Table 4, Figure 2). The antioxidant activity (ABST) was significantly correlated with TAC (0.933), Dp3-Sam (0.932), Dp3-Glu (0.924), and Cy3-Sam (0.913) contents (*p* < 0.001). The TPC, TFC, and GC contents also exhibited significant correlation coefficients ranging between 0.526 and 0.567 (*p* < 0.05). Consequently, the antioxidant activity in *H. acetosella* was strongly correlated with its contents of phenolic compounds, with the related anthocyanin contents also being involved in the antioxidant activity. In contrast, the antibacterial activity against Gram-positive bacteria was not significantly correlated with the content of phenolic compounds but antibacterial activity against Gram-negative bacteria was negatively correlated with the GAL content (−0.433, *p* < 0.05). However, the content of GAL is too low in *H. acetosella* to produce an antibacterial response. Overall, the antibacterial activity varied between the accessions, suggesting the involvement of other specific phenolic compounds present in their extracts. Borrás-Linares et al. [4] have also reported that the antimicrobial assay revealed no significant or a negative correlation between phenolic contents and antibacterial activity in *H. sabdariffa*, suggesting a similar response. Hierarchical clustering classified the accessions and measurements according to their chemical and biofunctional similarities. The accessions divided into two clusters: the first cluster contained high contents of anthocyanins and the second cluster according to the status of the phenolic compounds and biofunctional properties. The measurements were classified into three clusters: cluster I contained antibacterial activities, cluster II contained phenolic compounds without anthocyanins, and cluster III contained antioxidant activity with anthocyanins (Figure 2). These results, as mentioned earlier, indicated that the antioxidant activity was strongly associated with the levels of anthocyanins such as TAC, Dp3-Sam, Dp3-Glu, and Cy3-Sam.

## 3. Materials and Methods

### 3.1. Plant Materials

The 18 accessions studied (Table 1, Figure 1) were collected from the USDA and originated in Zambia. The leaves of the genotypes were harvested in August for each accession for the analysis of the functional compounds. The leaves were picked by hand from plants grown in three separate plots on the same plantation. The seeds were planted 20 cm apart in rows 60 cm apart in plots (3 × 4.2 m). Fertilizer (N:P:K 4:2:2 *w*/*w*/*w*) was applied at 550 kg/ha shortly after seeding. The experiment was conducted at the Korea Atomic Energy Research Institute (35°30′33.9″ N, 126°50′02.3″ E, Jeongeup, Korea). The plants’ cultivation conditions between May and August 2017 were as follows: mean temperature 17.8–24.4 °C, relative humidity 66.0–80.9%, mean sunlight 10.4–7.0 h. All of these climate data were accessed through the Korea Meteorological Administration web portal (http://weather.go.kr/).

### 3.2. Phenolic Compounds Extraction

All samples were ground to achieve a particle sizeable to pass through a 500-mesh sieve. The ground samples (1 g) were extracted in 5 mL of distilled water for 16 h then filtered through a 0.45-μm membrane filter. The antioxidant and antibacterial activities were measured using these aqueous extracts.

### 3.3. Total Phenolic Content

The total phenolic content (TPC) was determined by the Folin–Ciocalteu colorimetric method [7]. A small quantity (0.2 mL) of each extract and 1.5 mL of Folin–Ciocalteu reagent (20% *v*/*v*) were mixed thoroughly. Four mL of Na_2_CO_3_ (7%) were added, then made up to 10 mL with water. The mixture was kept in the dark at room temperature for 90 min. The absorbance was then measured at 760 nm using a UV-spectrophotometer (UV-1800, Shimadzu, Kyoto, Japan). TPC was calculated using a calibration curve of gallic acid.

### 3.4. Total Flavonoid Content

The total flavonoid content (TFC) of the *H. acetosella* extracts was determined as described by Ryu et al. [7]. Each extract sample (0.2 mL) was added to 4 mL double-distilled water and 0.3 mL of 5% NaNO_2_ in a flask. The samples were left for 5 min, then 0.3 mL of 10% AlCl_3_ was added. After 6 min, 2 mL NaOH were added then made up to 10 mL with double-distilled water. The absorbance was then measured at 510 nm. TFC was calculated using a calibration curve of quercetin equivalents.

### 3.5. Total Anthocyanin Content

The total anthocyanin contents (TAC) of the *H. acetosella* extracts were determined as described by Sutharut et al. [36]. The pH differential method, consisting of a KCl buffer (0.025 M, pH 1.0) and a CH_3_COONa buffer (0.4 M, pH 4.5), was used to determine the total anthocyanin content of the methanol extract prepared from each sample. A 1-mL aliquot of the extract was mixed with 4 mL of each of the buffers then incubated at 28 °C for 15 min so that the solution could equilibrate. The absorbance was measured at 510 nm and 700 nm using deionized water as a blank. The final result was converted to milligrams of cyanidin-3-glucoside equivalents (CGE) per gram dry weight (mg CGE/g dry weight).

### 3.6. UPLC Analysis

The phenolic compounds were analyzed using a ultra-high performance liquid chromatography (UPLC) system (CBM-20A, Shimadzu) with two gradient pump systems (LC-30AD, Shimadzu), a UV-detector (SPD-M30A, Shimadzu), an auto sample injector (SIL-30AC, Shimadzu), and a column oven (CTO-30A, Shimadzu). Separation was achieved on an XR-ODS column (3.0 × 100 mm, 1.8 μm, Shimadzu) using a linear gradient elution program with a mobile phase containing solvent A (0.1%, *v*/*v*, trifluoroacetic acid in distilled deionized water) and solvent B (0.1%, *v*/*v*, trifluoroacetic acid in acetonitrile). The phenolic compounds were separated using the following gradient: 0–5 min, 10–15% B; 5–10 min, 15–20% B; 10–15 min, 20–30% B; 15–25 min, 30–50% B; 25–30 min, 50–75% B; 30–35 min, 75–100% B; 35–40 min, 100–5% B; and 40–45 min, 5–0% B. The phenolic compounds and anthocyanins were detected at 280 nm and 520 nm, respectively. The chlorogenic acid (CGA), caffeic acid (CA), delphinidin-3-sambubioside (Dp3-Sam), delphinidin-3-glucoside (Dp3-Glu), cyanidin-3-sambubioside (Cy3-Sam), all obtained from Sigma-Aldrich Co. (St. Louis, MO, USA) were identified based on the retention times of commercial standards (UV spectrum). Gallocatechin (GC) and gallic acid (GAL) were identified as described in a previous study [16] and by their UV-visible spectral characteristics.

### 3.7. ABTS Radical Cation Scavenging Activity

Each extract sample was diluted 10-fold with water, then the ABTS value was evaluated as described by Re et al. [25]. In brief, ABTS was measured using pre-formed radical monocations. The mixtures, along with 7.4 mM ABTS solution and 2.6 mM potassium persulfate, were incubated at room temperature in the dark for 24 h. The ABTS solution was diluted with phosphate-buffered saline (pH 7.4) to achieve an absorbance of 0.7 ± 0.02 at 734 nm. Each sample (10 µL) was then reacted with 190 µL of the ABTS solution. The absorbance at 734 nm was measured 6 min after the reaction using the Benchmark Plus ELISA reader (Bio-Rad, Hercules, CA, USA).

### 3.8. Antibacterial Activity Assays

The antibacterial activities of the extracts were tested against two pathogenic species, a Gram-positive bacterium (*Staphylococcus aureus* ATCC 6538) and a Gram-negative bacterium (*Pseudomonas aeruginosa* ATCC 9027), using the agar-well diffusion method [4] with some modification. The two bacterial cultures were grown in Difco Nutrient Agar (BD Difco, Franklin Lakes, NJ, USA) for 24 h at 32 °C then diluted with sterile distilled water to obtain an inoculum concentration of 10^6^ cfu/mL before the suspensions were spread on the nutrient agar medium. Aliquots of each phenolic extract (70 μL) were added to an 8-mm diameter paper disc placed on the plates then incubated at 32 °C for 24 h. The positive and negative controls used gentamicin (5 μg) (Merck, Kenilworth, NJ, USA) and sterile distilled water, respectively. The antibacterial activity was determined as the diameter of the inhibition zones (mm) around the discs from each extract.

### 3.9. Statistical Analysis

The chemical analysis and biofunctional assay data were assessed using multiple variance analysis (ANOVA) with Duncan’s multiple range *post-hoc* test in the SPSS version 20 statistical software package (IBM Corp., Armonk, NY, USA). Differences between mean values were considered to be significant at *p* < 0.05. The hierarchical clustering analysis was performed using the complete linkage method based on the phenolic compound contents and biofunctional data from the 18 different *H. acetosella* accessions. The phenolic compounds were visualized as z-values on a heatmap.

## 4. Conclusions

In this study, the composition of phenolic compounds in the leaf extracts of 18 different *H. acetosella* accessions was determined and how it contributed to their antioxidant and antibacterial activities. The antioxidant activity was significantly associated with the level of anthocyanins in the phenolic extracts. This study is the first to report the antibacterial activity of *H. acetosella* against both Gram-positive and Gram-negative bacteria. These results could be useful for exploring the potential of medicinal crops as a valuable resource for discovering new pharmaceutical drugs.

## Figures and Tables

**Table 1:** Leaf and petiole color of the 18 different *H. acetosella* accessions used in this study.

Number	ID	Leaf Color	Petiole Color
1	PI 500707	Green	Green-red
2	PI 500730	Green	Green-red
3	PI 500744	Green	Green-red
4	PI 500749	Green	Green
5	PI 500755	Green	Green
6	PI 500756	Green	Green
7	PI 500758	Green	Green
8	PI 500761	Green	Red
9	PI 500764	Green	Light-red
10	PI 500765	Green	Light-red
11	PI 500766	Green	Green-red
12	PI 500777	Red	Red
13	PI 500778	Green	Green
14	PI 500794	Green	Green
15	PI 500801	Red	Red
16	PI 500804	Green	Green
17	PI 500805	Red	Red
18	PI 591552	Green	Green-red

**Table 2:** Contents of phenolic compounds in leaf extracts from 18 different *H. acetosella* accessions (mean value ± S.D., n = 3).

Accessions	TPC	TFC	TAC	GC	GAL	CGA	CA	Dp3-Sam	Dp3-Glu	Cy3-Sam
PI 500707	434.67 ± 16.0 ^a^*	262.19 ± 11.6 ^a^	0.47 ± 0.11 ^a^	1.57 ± 0.07 ^a^	1.97 ± 0.8 ^b^	41.56 ± 5.74 ^a^	42.93 ± 7.11 ^a^	Nd ^(1)^	nd	nd
PI 500730	297.52 ± 27.8 ^d^	229.69 ± 9.7 ^b^	0.44 ± 0.10 ^a^	0.95 ± 0.04 ^f^	0.67 ± 0.2 ^a^	13.2 ± 3.18 ^de^	27.72 ± 0.49 ^de^	nd	nd	nd
PI 500744	291.62 ± 23.8 ^de^	227.57 ± 7.0 ^bc^	0.35 ± 0.08 ^a^	0.96 ± 0.05 ^f^	0.86 ± 0.38 ^a^	8.66 ± 1.02 ^f^	30.5 ± 0.57 ^cd^	nd	nd	nd
PI 500749	283.58 ± 12.0 ^de^	222.03 ± 6.5 ^bc^	0.3 ± 0.06 ^a^	0.68 ± 0.04 ^h^	0.52 ± 0.12 ^a^	5.28 ± 0.59 ^fg^	35.83 ± 0.24 ^bc^	nd	nd	nd
PI 500755	282.72 ± 25.0 ^de^	210.71 ± 8.9 ^cd^	0.12 ± 0.03 ^a^	0.74 ± 0.03 ^gh^	0.91 ± 0.42 ^a^	18.45 ± 3.13 ^cd^	24.49 ± 0.09 ^ef^	nd	nd	nd
PI 500756	302.04 ± 23.1 ^d^	217.42 ± 7.2 ^bc^	0.13 ± 0.04 ^a^	0.77 ± 0.04 ^gh^	0.87 ± 0.4 ^a^	16 ± 3.07 ^d^	31.35 ± 0.24 ^cd^	nd	nd	nd
PI 500758	288.29 ± 29.6 ^de^	210.27 ± 8.7 ^cd^	0.15 ± 0.05 ^a^	0.83 ± 0.05 ^fg^	0.98 ± 0.44 ^a^	21.25 ± 3.21 ^cd^	35.03 ± 7.74 ^bc^	nd	nd	nd
PI 500761	248.2 ± 21.9 ^ef^	219.24 ± 10.3 ^bc^	0.59 ± 0.15 ^a^	1.07 ± 0.07 ^de^	0.63 ± 0.14 ^a^	5.4 ± 1.19 ^fg^	22.79 ± 0.14 ^ef^	nd	nd	nd
PI 500764	230.94 ± 7.3 ^g^	199.1 ± 9.7 ^f^	0.42 ± 0.08 ^a^	0.73 ± 0.03 ^gh^	0.59 ± 0.13 ^a^	9.66 ± 2.38 ^ef^	24.93 ± 1.09 ^ef^	nd	nd	nd
PI 500765	378.5 ± 13.6 ^b^	224.32 ± 8.3 ^bc^	0.43 ± 0.08 ^a^	0.8 ± 0.05 ^gh^	1.05 ± 0.52 ^a^	35.25 ± 1.08 ^b^	32.23 ± 0.32 ^cd^	nd	nd	nd
PI 500766	249.77 ± 27.6 ^ef^	203.53 ± 14.2 ^d^	0.32 ± 0.10 ^a^	1.14 ± 0.10 ^cd^	0.94 ± 0.48 ^a^	17.35 ± 1.49 ^cd^	14.95 ± 1.83 ^f^	nd	nd	nd
PI 500777	409.81 ± 37.6 ^ab^	254.03 ± 4.9 ^a^	19.98 ± 4.5 ^b^	1.15 ± 0.07 ^cd^	1.13 ± 0.61 ^a^	18.37 ± 1.62 ^cd^	38.28 ± 2.46 ^ab^	9.37	0.65	2.00
PI 500778	276.25 ± 30.0 ^de^	211.46 ± 7.8 ^cd^	0.1 ± 0.03 ^a^	0.78 ± 0.02 ^gh^	0.85 ± 0.37 ^a^	16.53 ± 3.01 ^d^	35.59 ± 7.81 ^bc^	nd	nd	nd
PI 500794	289.39 ± 15.1 ^de^	207.26 ± 9.3 ^d^	0.17 ± 0.04 ^a^	0.69 ± 0.02 ^h^	0.87 ± 0.39 ^a^	21.7 ± 3.02 ^c^	26.58 ± 0.44 ^de^	nd	nd	nd
PI 500801	309.72 ± 15.6 ^d^	227.99 ± 6.8 ^bc^	17.25 ± 3.7 ^b^	1.29 ± 0.13 ^b^	0.95 ± 0.51 ^a^	5.7 ± 0.25 ^fg^	32.01 ± 0.56 ^cd^	8.20	0.42	1.88
PI 500804	235.17 ± 19.5 ^g^	204.19 ± 9.3 ^d^	0.16 ± 0.05 ^a^	0.95 ± 0.09 ^f^	0.82 ± 0.36 ^a^	10.2 ± 2.63 ^ef^	21.87 ± 0.07 ^ef^	nd	nd	nd
PI 500805	360.67 ± 20.6 ^c^	227.65 ± 9.7 ^bc^	18.3 ± 4.0 ^b^	1.26 ± 0.13 ^bc^	1.11 ± 0.59 ^a^	20.49 ± 1.69 ^cd^	31.67 ± 0.46 ^bc^	9.02	0.46	1.68
PI 591552	193.14 ± 14.0 ^h^	199.74 ± 12.6 ^df^	0.42 ± 0.12 ^a^	1.01 ± 0.10 ^ef^	0.73 ± 0.36 ^a^	3.02 ± 0.42 ^h^	16.64 ± 0.61 ^e^	nd	nd	nd

Phenolic compounds (mg/100 g): total phenolic contents (TPC), total flavonoid contents (TFC), total anthocyanin contents (TAC), gallocatechin (GC), gallic acid (GAL), chlorogenic acid (CGA), caffeic acid (CA); anthocyanins (mg CGE/g): delphinidin-3-sambubioside (Dp3-Sam), delphinidin-3-glucoside (Dp3-Glu), cyanidin-3-sambubioside (Cy3-Sam). * The letters adjacent to mean value indicate the result of Duncan’s multiple range test at the 5% probability level (n = 3); ^(1)^ not detectable.

**Table 3:** Antioxidant and antibacterial activities of 18 different *H. acetosella* accessions.

ID	Antioxidant Activity	Antibacterial Activities (mm) ^(2)^	
	ABTS (%)	*S. aureus*	*P. aeruginosa*
Control ^(1)^	99.83 ± 0.18	12.80 ± 0.34	13.10 ± 0.42
PI 500707	65.94 ± 0.18 ^h^	12.00 ± 1.00 ^a^	11.33 ± 0.58 ^ab^
PI 500730	59.36 ± 0.13 ^bc^	12.33 ± 0.58 ^ab^	13.00 ± 0.00 ^de^
PI 500744	64.47 ± 0.18 ^fg^	13.33 ± 0.58 ^bc^	12.33 ± 0.58 ^cd^
PI 500749	62.25 ± 0.22 ^de^	12.00 ± 0.00 ^a^	12.00 ± 0.00 ^bc^
PI 500755	59.44 ± 0.99 ^bc^	12.00 ± 0.00 ^a^	12.00 ± 0.00 ^bc^
PI 500756	57.47 ± 0.15 ^a^	13.00 ± 0.00 ^abc^	12.00 ± 0.00 ^bc^
PI 500758	62.46 ± 0.15 ^de^	13.67 ± 0.58 ^c^	11.33 ± 0.58 ^ab^
PI 500761	65.10 ± 0.15 ^gh^	13.33 ± 0.58 ^bc^	13.33 ± 0.58 ^e^
PI 500764	61.91 ± 0.23 ^d^	13.67 ± 0.58 ^c^	12.00 ± 0.00 ^bc^
PI 500765	64.05 ± 0.36 ^f^	12.67 ± 0.58 ^abc^	12.00 ± 0.00 ^bc^
PI 500766	60.11 ± 0.51 ^c^	12.00 ± 0.00 ^a^	11.67 ± 0.58 ^bc^
PI 500777	82.34 ± 0.23 ^j^	12.67 ± 0.58 ^abc^	11.33 ± 0.58 ^ab^
PI 500778	61.75 ± 0.40 ^d^	12.33 ± 0.58 ^ab^	12.00 ± 0.00 ^bc^
PI 500794	63.00 ± 0.38 ^e^	12.67 ± 0.58 ^abc^	11.67 ± 0.58 ^bc^
PI 500801	74.71 ± 0.07 ^i^	12.67 ± 0.58 ^abc^	11.67 ± 0.58 ^bc^
PI 500804	58.56 ± 0.08 ^b^	13.33 ± 0.58 ^bc^	12.00 ± 0.00 ^bc^
PI 500805	84.02 ± 0.15 ^k^	13.33 ± 0.58 ^bc^	11.33 ± 0.58 ^ab^
PI 591552	65.61 ± 0.08 ^h^	12.67 ± 0.58 ^abc^	10.67 ± 0.58 ^a^

^(1)^ Control used were: ascorbic acid (500 µM) in antioxidant assay and gentamicin (5 μg) (antibacterial assay). ^(2)^ Inhibition zone of *H. acetosella* leaf extract against *Staphylococcus aureus* (Gram-positive) and *Pseudomonas aeruginosa* (Gram-negative). The results are shown as the mean ± standard error of three replicates. Mean with the same letter are not significantly different at the 5% probability level (Duncan’s multiple range test). nd; not detectable.

**Table 4:** Correlation coefficients between the contents of phenolic compounds and antioxidant/bacterial activities.

	TPC	TFC	TAC	GC	GAL	CGA	CA	Dp3-Sam	Dp3-Glu	Cy3-Sam	ABST	*S. aureus*	*P. aeruginosa*
TPC	1.000	0.887 ***	0.475 *	0.478 *	0.770 ***	0.750 ***	0.787 ***	0.471 *	0.499 *	0.462 *	0.528 *	−0.233	−0.175
TFC		1.000	0.470 *	0.636 **	0.685 **	0.475 *	0.747 ***	0.458 *	0.498 *	0.463 *	0.526 *	−0.265	−0.024
TAC			1.000	0.508 *	0.223	−0.040	0.307	1.000 ***	0.989 ***	0.997 ***	0.933 ***	0.101	−0.317
GC				1.000	0.693 **	0.258	0.199	0.501 *	0.475 *	0.501 *	0.567 *	−0.117	−0.250
GAL					1.000	0.811 ***	0.532 **	0.223	0.228	0.216	0.317	−0.254	−0.433 *
CGA						1.000	0.491 *	−0.037	−0.021	−0.058	0.063	−0.255	−0.245
CA							1.000	0.306	0.328	0.309	0.349	−0.095	−0.115
Dp3-Sam								1.000	0.986 ***	0.996 ***	0.932 ***	0.106	−0.323
Dp3-Glu									1.000	0.986 ***	0.924 ***	0.084	−0.325
Cy3-Sam										1.000	0.913 ***	0.086	−0.315
ABST											1.000	0.145	−0.380 *
*S. aureus*												1.000	0.090
*P. aeruginosa*													1.000

Significant levels are indicated as; * (0.05 > *p* > 0.01), ** (0.01 > *p* > 0.001), *** (0.001 > *p*).

## Supplementary Materials

The Supplementary Materials are available online. Figure S1: Ultra-high performance liquid chromatography (UPLC) 3D profiles of the 18 *H. acetosella* accessions. The numbers in the boxes correspond to the numbers in column 1 of Table 1. Figure S2: Agar-well diffusion test demonstrating inhibition zones against *S. aureus* and *P. aeruginosa* bacteria. All test were performed three replicates. Table S1: DPPH radical activity of 18 different *H. acetosella accessions*.
