# Supplementary material for: Phenolic Compounds in Extracts of Hibiscus acetosella (Cranberry Hibiscus) and Their Antioxidant and Antibacterial Properties
Source: Molecules. 2020 Sep 12;25(18):4190. doi: 10.3390/molecules25184190 (PMC7571108; doi:10.3390/molecules25184190)
Supplement: Supplementary file 1 [file molecules-25-04190-s001.zip › Table S1.docx]

Table S1. DPPH radical activity of 18 different *H. acetosella* accessions

|  |  |  |
| --- | --- | --- |
| ID | | DPPH activity  (%) |
| PI 500707 | | 37.61 ± 2.61 |
| PI 500730 | | 28.51 ± 8.71 |
| PI 500744 | | 90.59 ± 1.75 |
| PI 500749 | | 89.60 ± 0.81 |
| PI 500755 | | 87.21 ± 3.74 |
| PI 500756 | | 89.47 ± 2.02 |
| PI 500758 | | 88.10 ± 2.89 |
| PI 500761 | | 92.94 ± 3.36 |
| PI 500764 | | 92.19 ± 3.07 |
| PI 500765 | | 90.29 ± 0.53 |
| PI 500766 | | 89.86 ± 1.71 |
| PI 500777 | | 119.21 ± 0.24 |
| PI 500778 | | 91.84 ± 0.48 |
| PI 500794 | | 88.42 ± 1.31 |
| PI 500801 | | 86.27 ± 9.28 |
| PI 500804 | | 85.70 ± 1.24 |
| PI 500805 | | 109.53 ± 4.67 |
| PI 591552 | | 82.82 ± 1.11 |
