# Supplementary figures and images for: Phenolic Compounds in Extracts of Hibiscus acetosella (Cranberry Hibiscus) and Their Antioxidant and Antibacterial Properties
Source: Molecules. 2020 Sep 12;25(18):4190. doi: 10.3390/molecules25184190 (PMC7571108; doi:10.3390/molecules25184190)

Fig. S1.

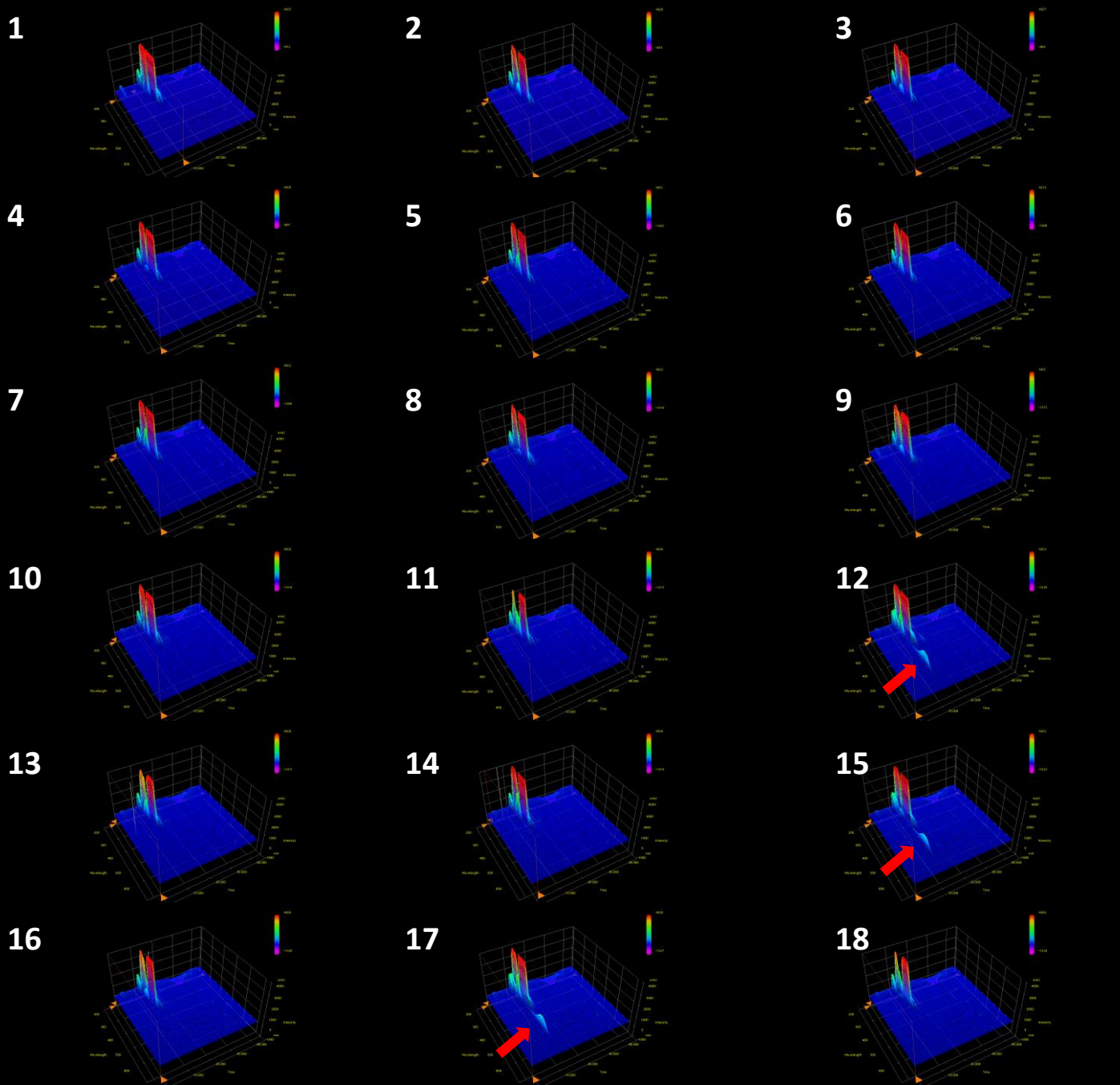

Supplement: Supplementary file 1 [file molecules-25-04190-s001.zip › Fig.S1_UPLC_3D profiles.pdf]

Fig. S2

*Staphylococcus aureus*

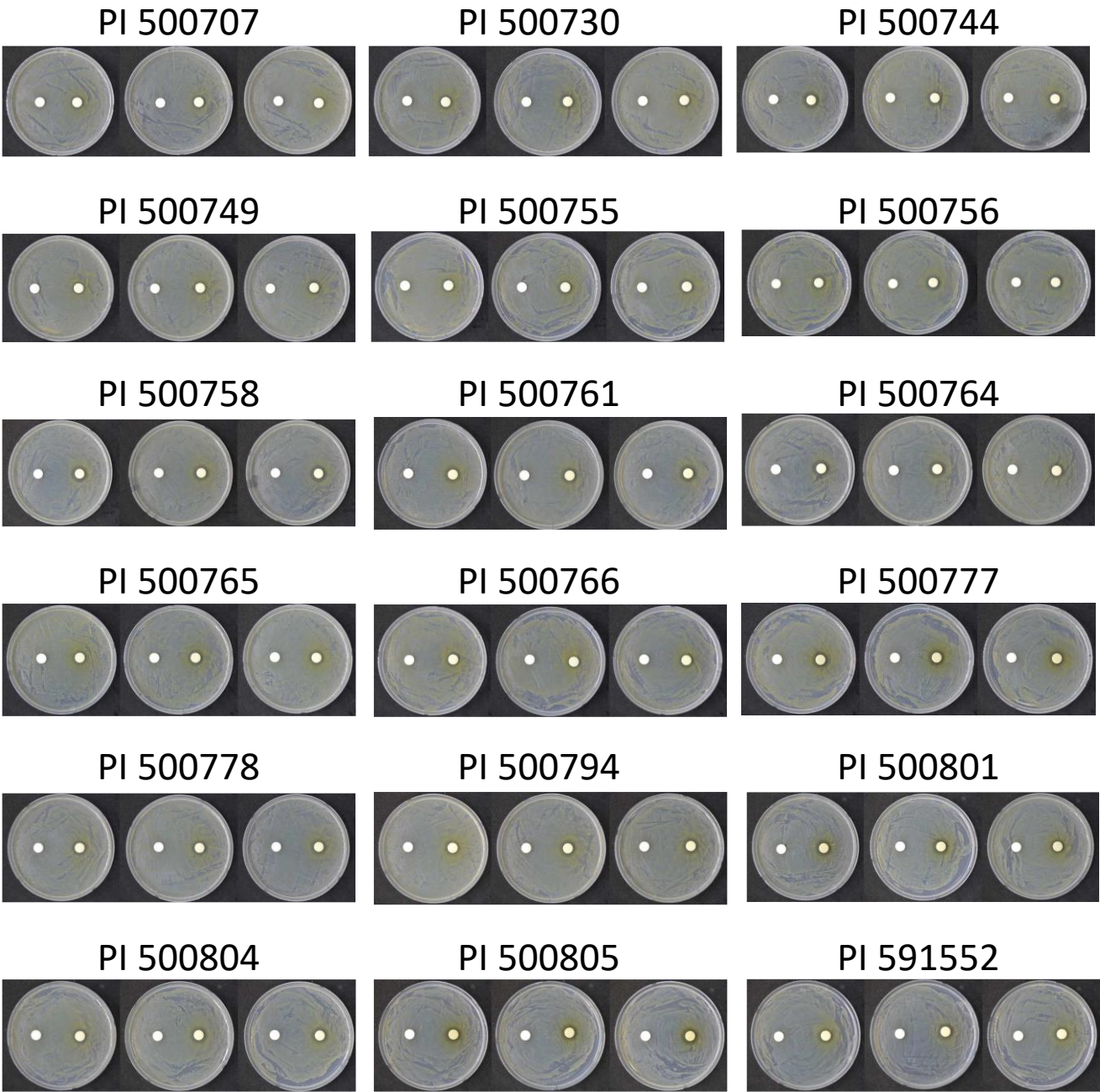

Fig. S2 continued

*Pseudomonas aeruginosa*

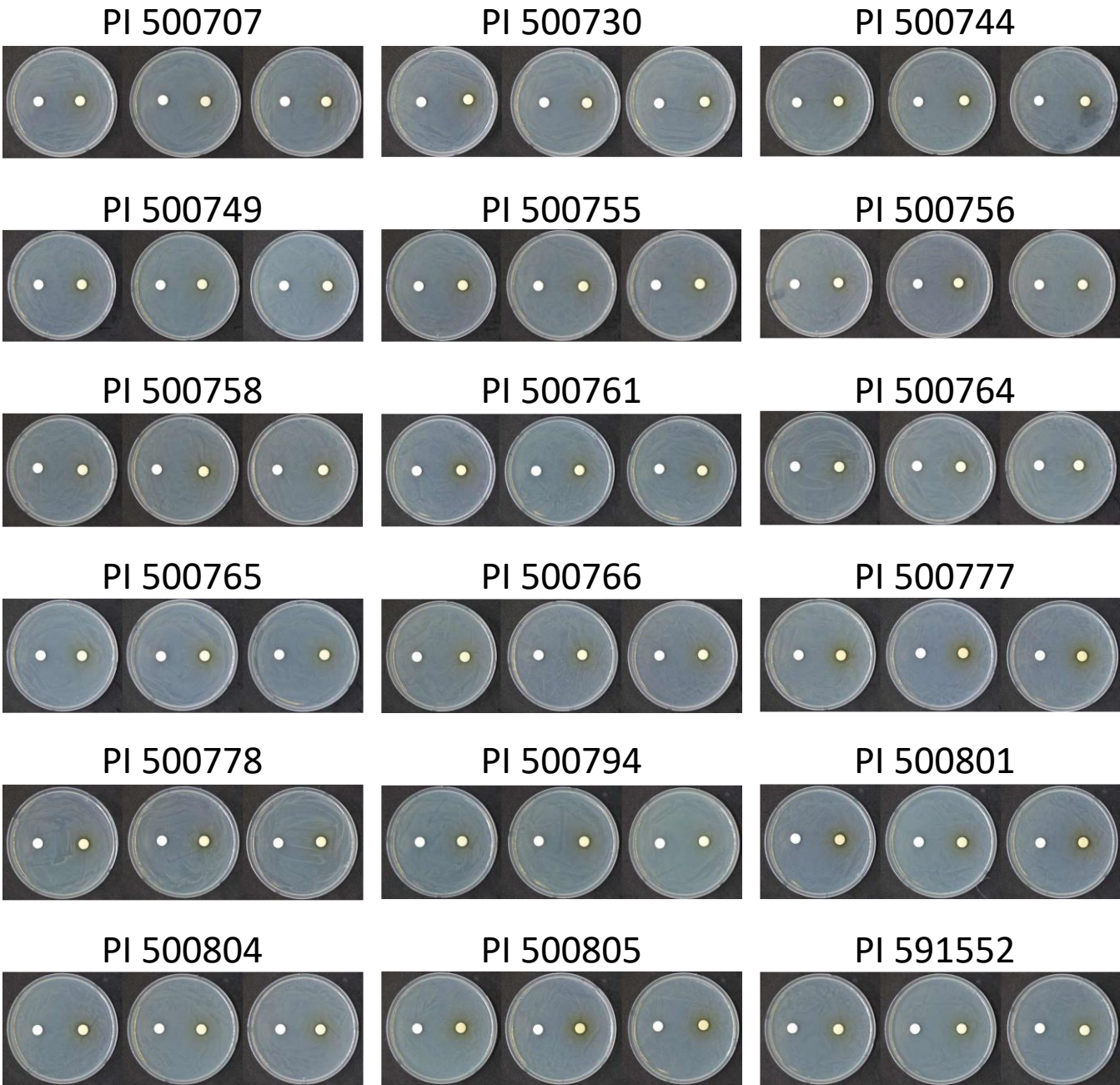

Supplement: Supplementary file 1 [file molecules-25-04190-s001.zip › Fig.S2_agar-well diffusion assay.pdf]
